# Review of the New World *Notomicrus* Sharp (Coleoptera, Noteridae) I: Circumscription of species groups and review of the *josiahi* group with description of a new species from Brazil

**DOI:** 10.3897/zookeys.1025.60442

**Published:** 2021-03-22

**Authors:** Stephen M. Baca, Andrew Edward Z. Short

**Affiliations:** 1 University of Kansas, Department of Ecology and Evolutionary Biology, Lawrence KS, USA University of Kansas Lawrence United States of America; 2 University of Kansas, Biodiversity Institute, Division of Entomology, Lawrence KS, USA University of Kansas Lawrence United States of America

**Keywords:** Aquatic beetles, Brazil, new species, South America, taxonomy

## Abstract

The New World species of the minute aquatic beetle genus *Notomicrus* Sharp compose a much greater diversity than their Old World congeners, with 14 of the 17 known *Notomicrus* species occurring in the Neotropics. A recent phylogenetic study recovered four primary New World species groups and found that there are a number of undescribed species across all of these main lineages. Here, we provide a taxonomic key to these New World species groups, including two described species that we currently do not place in any group (“*incertae sedis*” species), complete with images and illustrations of diagnostic characters and taxonomic notes including a list of known species in each group. This work provides a scaffold for further planned taxonomic revisions within the genus. In addition, we review the first of the four New World groups, the *josiahi* species group and describe one new taxon, *N. interstinctus***sp. nov.** from northern Brazil. Provided are descriptions, habitus images and illustrations of diagnostic characters.

## Introduction

*Notomicrus* Sharp is the most speciose genus of the minute aquatic beetle subfamily Notomicrinae (Coleoptera: Noteridae). Its distribution spans Indomalaya, Oceania and the New World, though the majority of *Notomicrus* diversity occurs in the Neotropics (14 of 17 described species). *Notomicrus* species occupy a wide range of habitats, including the margins of ponds, streams, marshes and swamps, drying stream beds, forest pools, hygropetric habitats and terrestrial leaf litter. Some species present a high specificity in their habitat preference, while others are found to be more generalists (Baca and Short 2020; personal observation). This ecological plasticity is a quality of the subfamily Notomicrinae as a whole. Both of the other notomicrine genera are subterranean specialists: *Phreatodytes* Uéno from aquifers in Japan and *Speonoterus* Spangler, a monotypic genus known only from a single collection in a shallow cave in Indonesia. *Speonoterus* appears to be a very close relative of *Notomicrus*, with *Phreatodytes* being their sister-group (Baca et al. 2017; Baca and Short 2020). It has been speculated that, given the plasticity of the habit preferences of *Notomicrus* and the aforementioned morphological similarity, *Speonoterus* may represent a specialized *Notomicrus* species (Baca and Short 2020). These relationships remain to be tested with molecular sequence data as *Speonoterus* is known only from the few specimens of the type series (Spangler 1996).

Since its establishment by Sharp (1882), the classification of *Notomicrus* and its species has been very stable. *Notomicrus nanulus* (LeConte, 1863) and *Notomicrus tenellus* (Clark, 1863) are the only species that have required nomenclatural recombination as these were described before the genus was established, thus necessitating transfer (Nilsson 2011; note that some synonyms of *N. tenellus* were also described before the erection of *Notomicrus*). Few junior synonyms of any *Notomicrus* species have been described (Nilsson 2011), and the genus itself has no current generic synonyms. Classification changes have occurred at higher levels, for example, tribe, subfamily and family levels, but the genus status of *Notomicrus* has remained unaltered.

The monophyly of *Notomicrus* has been previously supported, with the Old and New World clades each also being found to represent reciprocally monophyletic lineages (Baca et al. 2017; Baca and Short 2020). These studies have also revealed that, unsurprisingly, there remain many undescribed species in the genus, especially in South America. This is further indicated by recent descriptions of new Neotropical species (Miller 2013; Manuel 2015; Baca and Short 2018; Guimarães and Ferreira-Jr 2019). Together, these works have greatly strengthened our understanding of notomicrine diversity and evolution. However, in effect, Young’s (1978) benchmark revision of the New World *Notomicrus* now includes just over half of the currently described species, amplifying the need for a comprehensive treatment of the group, especially with more diversity remaining to be described.

The species-level phylogenetic reconstruction of Baca and Short (2020) placed heavy emphasis on New World *Notomicrus* diversity. They recovered New World *Notomicrus* as diverging into four clades, the *josiahi*, *nanulus*, *meizon* and *traili* species groups, reciprocally supported by morphological characters. As such, Baca and Short (2020) provide an appropriate scaffold for taxonomic treatment of the groups.

Here, we (1) diagnose and provide a taxonomic key to the four primary species groups of New World *Notomicrus*. As part of this objective, we review morphological characters of importance, illustrate diagnostic characters and provide habitus images of exemplar species, taxonomic notes and a list of known species and references for each group. We then (2) present the first of four species-level revisionary works of New World *Notomicrus* by reviewing the *josiahi* species group. Included are a diagnosis of the group, a re-description of *N. josiahi* Miller, 2013 and a description of a new species from Brazil.

## Materials and methods

### Observations and measurements

Specimens were observed and measured using an Olympus SZX7 stereomicroscope. The microscope was equipped with 10× eyepieces, a DF PL 2×_-4_ objective (16–112× magnification) and a calibrated ocular micrometer. Genitalia and tarsal claws were relaxed in hot water and dissected. Dissections were placed in glycerine on glass slides for observation. For additional observations and images of the prosternal process, aedeagi and tarsal claws, selected specimens were cleared in a warm 10% potassium hydroxide (KOH) solution and periodically checked multiple times an hour. Once desired elimination of soft tissue was achieved, specimens were thoroughly rinsed in DI (deionized) water. In some cases, DNA voucher specimens were used for observation and imaging of structures as the lysing process also dissolves soft tissue, effectively clearing the specimen and negating the need to damage additional specimens.

### Images and illustrations

Dorsal habitus images were obtained with a Visionary Digital microphotography system equipped with an Infinity K2 microscope using a 5× objective and Helicon Focus imaging software. Photos were aligned and stacked using CombineZP (www.hadleyweb.pwp.blueyonder.co.uk) and refined in Adobe Photoshop. Ventral images and images of structures to be used for illustrations were taken with an Olympus DP72 camera system attached to either an Olympus SZX16 stereomicroscope with an SDF PLAPO 1×PF or 2×PF objective or an Olympus BX51 compound microscope with an UPlanFLN 40× oil immersion objective. The digital images were then stacked as above, with structures traced using Adobe Illustrator. Prolegs, prosterna, noterid platforms and male genitalia were imaged with the aforementioned stereomicroscope imagining system; illustrations were traced from these images. Male genitalia were placed in a depression slide with a drop of KY jelly and the remainder of the depression was filled with ethanol (EtOH). The KY jelly maintains its viscosity so that genitalia will hold its position for imaging. The EtOH eliminates obscuring refraction. Tarsal claws were imaged on the compound microscope. The fifth (V) pro- and metatarsomeres with tarsal claws were placed on a flat slide with EtOH and a cover slip was applied and glycerine was then used the seal the outside of the slip. The lower surface tension of the EtOH allows the cover slip to press on the claws, flattening them against the slide.

### Terminology

Descriptive terminology follows previous works (e.g. Manuel 2015; Baca and Short 2018).

**Noterid platform**. In *Notomicrus*, the noterid platform is formed by the raised projections of the inner metacoxal lamellae.

**Genitalia and appendages**. Following Miller and Nilsson (2003), genitalia and appendages are described in their fundamental homologous positions.

### List of depositories

**INPA**Instituto Nacional de Pesquisas da Amazônia, Manaus, Brazil (N. Hamada);

**MIZA** Museo del Instituto de Zoología Agrícola, Maracay, Venezuela (L. Joly);

**MSB**Museum of Southwestern Biology, University of New Mexico (K. Miller);

**NHM**Natural History Museum, London, UK (M. Barclay, C. Taylor);

**SEMC** Snow Entomological Collection, University of Kansas, Lawrence, KS (A. Short);

**USNM**U.S. National Museum of Natural History, Smithsonian Institution, Washington, DC (C. Micheli).

### Structures of taxonomic importance for diagnoses of *Notomicrus* species

**Size.** The total body length of *Notomicrus* species ranges between ca. 1.0 mm and 1.8 mm. Following Young (1978), size can, in combination with other characters, be very helpful in species determination. Size is quantified in terms of (1) total length (TL), as measured from anterior margin of head to apex of elytra, in dorsal aspect, (2) total length without head (TLPn), measured from medial anterior margin of pronotum to elytral apex (this is included to provide a consistent length measurement, as the degree to which the head is ventrally reflexed can affect the TL measurement) and (3) greatest width (GW), as measured transversely at the widest point of the beetle. Means of the measurements for each species, with standard deviations (SD) of the mean are also presented. Ratios of TL and GW are given as a way of quantifying the shape of the body outline. Means of the measurements for each species, with standard deviations (SD) of the mean for TL are also presented. Ratios of TL and GW are given as a way of quantifying the shape of the body outline.

**Figure 1.**
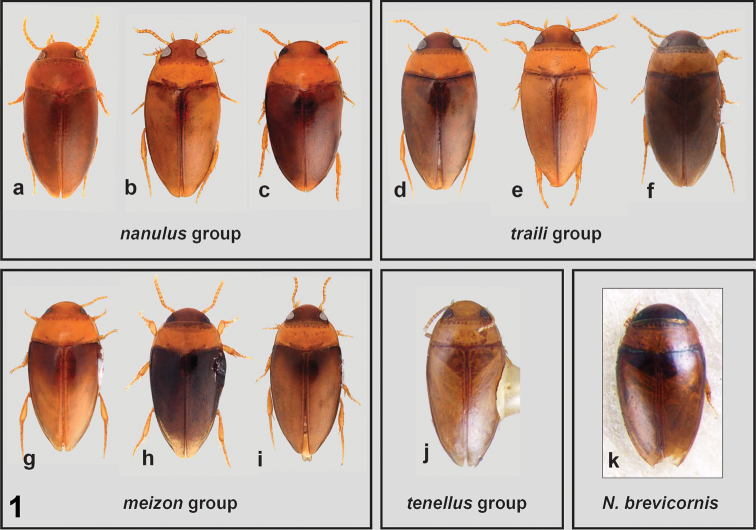
*Notomicrus* species groups. Dorsal habitus of representative *Notomicrus* species **a***N. nanulus***b***N. sharpi***c***N.* sp. **d**N. cf. traili**e**N. cf. gracilipes**f***N. petrareptans***g***N.* sp. **h**N. sp nr. malkini**i**N. sp. nr. meizon**j***N. tenellus***k***N. brevicornis* male syntype.

**Color.** Most species of *Notomicrus* present dorsal coloration as varying shades from brown to yellow. However, individuals of some species present specific color patterns among sclerites. For example, some species appear bicolorous, with the elytra and head darker brown and contrasting against a lighter colored pronotum, for example, *N. traili* Sharp, 1882 (Fig. 1d, f). Other species, such as *N. nanulus* (LeConte, 1863) (Fig. 1a–c), are more uniformly brown, with little contrast between elytra, head and pronotum. Others still, such as *N. josiahi* Miller, 2013, present elytra with dark areas distinctly contrasting against lighter areas and/or may have a notable iridescent sheen (Fig. 5). Color patterns of the ventral surface can also be helpful in delimiting species. Color is best used in conjunction with other characters, as many species share similar coloration. Intraspecific variation is often present, with individuals appearing relatively lighter or darker in color, this variation being additionally present between mature and teneral individuals.

**Punctation.** Elytral punctation can be very helpful in diagnosing species of *Notomicrus*. Many species differ in the relative coarseness, density and patterns of punctation. Punctation should be used in combination with other characters to diagnose species as this character often presents similarly across multiple species.

**Microsculpture.** External microsculpture in *Notomicrus* varies among species and, in combination with other characters, can be helpful for diagnosis. In *Notomicrus*, the microsculpture consists of a microreticulation, where a superficially impressed mesh of very fine lines or grooves creates small cells. This is usually present on most external sclerites of the head, thorax, abdomen and legs, though it may not be uniform across these sclerites in an individual (e.g. the microsculpture of the noterid platform often differs from that of the elytra). In particular, the degree of impression and size or density of the meshes can be characteristic for a species or group of species.

**Eye size.** The size of the eyes, relative to the head capsule, can be very useful in identifying species. Here, the relative eye size is presented as a ratio of the greatest width of the head (HW) and interocular distance (EW). Measurements are taken from dorsal aspect, approximately at posterolateral margins of the eyes. Interocular distance is taken from the narrowest point between the eyes. The larger the eyes relative to the head capsule, the larger the ratio HW/EW, for example, *N. josiahi*HW/EW = 2.35–2.53, *N. petrareptans* Baca & Short, 2018 HW/EW = 1.65–1.73.

**Prosternal process.** The shape of the prosternal process was observed to be variable among some species and species groups of *Notomicrus* (Fig. 3). In particular, the shape of the apex and degree of constriction between the procoxae can be diagnostic in combination with other characters. For example, being acutely angled as in *N. josiahi* (Fig. 3a) or more rounded or blunt, as in *N. nanulus* (Fig. 3b).

**Tarsal claws.** The pro- and mesotarsal claws of males of *Notomicrus* show significant interspecific variation in size and shape. Following Young (1978), the shape of the claws, as well as the relative sizes of the anterior claws and posterior claws can be helpful in diagnosing species. Here we describe and illustrate the claws in lateral view (Figs 8, 9). The finer details of the claws’ shape may be difficult to view without the use of a compound microscope. It should be noted that slide mounting the claws can variably alter the appearance compared to the *in situ* appearance under a stereomicroscope, this in part being due to their asymmetrical shape or the claws being slightly splayed on dried specimens. Characters of the tarsal claws are best used in combination with other characters.

**Aedeagus.** The aedeagus is especially helpful for diagnosing species. The median lobe should be observed from several angles as it tends to be asymmetric and an oblique orientation can give the appearance of a different shape. Despite relative reliability, the aedeagus is still best used in combination with other characters for identification. Many species, even those across species groups, can present very similar aedeagi. For example, the aedeagus of *N. interstinctus* sp. nov. (Fig. 7) converges very closely on members of the *traili* group. Additionally, the males of some species are unknown, suggesting these lineages may only comprise females, for example, *N. femineus* Manuel, 2015.

## Taxonomy

### Genus Notomicrus

Taxon classificationAnimaliaColeopteraNoteridae

Sharp, 1882

27F198FF-5540-58E6-8D1F-280EE7AEE712

#### Type species.

*N. brevicornis* Sharp, 1882. Designation by Guignot 1946: 115.

#### Diagnosis.

(1) Eyes present; (2) metacoxae and metaventrite fused, suture indistinct laterad of noterid platform; (3) noterid platform not extending anteriorly on to metaventrite; (4) protibia with loose rows of spines and setae, lacking large spur at apex and tight comb of small spines on distolateral margin and not expanded distally beyond protarsal insertion; (5) partial fusion of metafurca and metacoxae, not forming complete ring; (6) mid-gular apodeme absent (Beutel and Roughley 1987; Miller 2009); (7) female laterotergite short, posteriorly extending beyond base of gonocoxae (Miller 2009).

#### Remarks.

As noted by Miller (2009) and others (Manuel 2015 and citations therein), the characters that define *Notomicrus* are primarily plesiomorphic with the exception of the fusion of the metacoxae and metaventrite. *Speonoterus* Spangler is also defined by the above character combination, except absence of eyes. Spangler (1996) also noted that the distance from the anterior terminus of the noterid platform (metacoxal lamellae) to the mesocoxal cavities is shorter in *Speonoterus*, less than the width of the mesocoxal cavities, whereas in *Notomicrus*, this distance is greater than the width of the mesocoxal cavities (See Spangler 1996; Manuel 2015). Notomicrine species are all notably small (ca. 1.0–1.8 mm). Characters listed above without specific citation have been more common in use for defining *Notomicrus* (e.g. Sharp 1882; Young 1978; Buetel and Roughley 1987); see Miller (2009) and Manuel (2015) for details.

### Key to species groups and insertae sedis species of *Notomicrus* Sharp

This key is intended to be used as a first step in identifying New World species of *Notomicrus*. Identification of *Notomicrus* species can prove difficult for non-specialists, especially without additional species in hand for comparisons. Diagnoses of the species groups of *Notomicrus* also reflect this difficulty.

**Table:** 

1	Size small, TL = 1.3 mm. Elytral punctation almost entirely indistinct, except discal row and submargin of elytral suture with distinct punctures, with very fine scattered setose punctures near lateral margins; elytral surface with microreticulation consisting of round, isodiametric cells, somewhat scale-like in appearance. Head appendages short, antennomeres VI–X wider than long; apical palpomeres distinctly bifurcate with enlarged sensory fields. Aedeagus as in Fig. 2 of Guimarães and Ferreira-Jr (2019); median lobe with large base and very large processes and hooks. Male pro- and mesotarsal claws short, anterior protarsal claw expanded at base. Known only from high elevation hygropetric habitats in Minas Gerais, Brazil	***N. teramnus***
–	Size variable, ca. 1.2–1.9 mm. Elytral punctation and microsculpture variable. Antennomeres usually longer than wide; apical palpomeres variable. Median lobe of aedeagus without conspicuous hooks or large processes (e.g. Figs 6, 7). Male pro- and mesotarsal claws variable. Habitat preference variable	**2**
2	Dorsal (outer) margin of protibia without notable robust seta at or near mid-length (Fig. 2a). Eyes large relative to head capsule (Fig. 5), HW/EW ≥ 2.0. Elytra with notable contrasting dark and light colors (Fig. 5a, c). Profemur with > 3 distinct closely spaced setae on anteroventral margin (Fig. 2a); posteroventral margin of male profemur lacking notable protuberance, only weakly angled near mid-length (Fig. 2a); male protarsal claws very small, distinctly shorter than half the length of protarsomere V, anterior protarsal claw bifurcate, branching dorsally (Figs 8, 9)	***josiahi* group**
–	Dorsal margin of protibia with a robust seta at or near mid-length (often two in females), at least as long as most dorsal seta on dorsoapical angle (Fig. 2b–f). Eyes smaller, HW/HW < 2.0. Elytra with or without contrasting colors. Profemur with < 3 closely-spaced setae on anteroventral margin; posteroventral margin of male profemur with notable protuberance or acute angle at ca. mid-length; male protarsal claws variable, anterior claw length almost always at least half the length of protarsomere V, almost always larger than female claws, sometimes bifurcate	**3**
3	Noterid platform with angles of posterior lobes squared or rounded (Fig. 4b–e)	**4**
–	Noterid platform with angles posterior lobes acutely angled (as in Fig. 4a, f–l)	**5**
4	Elytral surface impunctate to weakly punctate, punctures usually inconspicuous and sporadic under normal magnification, except for discal series; microreticulation variably impressed, consisting of small, round, isodiametric cells, giving the appearance of small scales. Body form variable, but usually oblong, less attenuated posteriorly (Fig. 1a–c). Elytral color uniform, brown, sometimes shiny, not iridescent or only weakly so	***nanulus* group**
–	Punctation distinctly present and often dense on posterior half of elytra, punctures finely to moderately impressed, bearing short setae, often extending on to anterior half of elytra; microreticulation variably impressed, consisting of fine mesh-like reticulation. Body form variable, but more elongate and attenuated posteriorly (Fig. 1g–i). Color variable, but elytra of mature specimens of most species with darker triangular area medially at base (Fig. 1g, i); in most species, dorsal surface very shiny and iridescent	***meizon* group (in part)**
5	Color uniformly brown. Elytral surface with microreticulation variably impressed, consisting of small, round, isodiametric cells, giving the appearance of fine scales, somewhat shiny, but never iridescent; punctation variable. Males with anterior protarsal claw bifurcate or branched (as in Fig. 8a)	**6**
–	Color variable, uniform or bicolorous. Elytral surface with microreticulation variably impressed, consisting of fine mesh-like reticulation, sometimes iridescent. Males with protarsal claws never bifurcated or branched	**7**
6	Body form oblong, rounded posteriorly (as in Fig. 1l or similar to *nanulus* group, for example, Fig. 1d). Elytral surface weakly punctate. Median lobe in lateral view as in Fig. 12b. New World	***N. brevicornis* Sharp, 1882**
–	Body form ovoid, more elongate, more attenuated posteriorly (as in Fig. J). Elytral surface weakly to moderately punctate. Median lobe different. Indomalaya and Oceania	***tenellus* group**
7	Protibia with robust seta of dorsal margin distinctly distad of half-length of outer margin, approximately at 2/3 margin length (Fig. 2e), distance between robust seta and dorsoapical angle distinctly shorter than distance between robust seta and first seta from protibial insertion. Dorsal coloration uniformly brown or bicolorous (Fig. 1d–f), with pronotum distinctly lighter than head and elytra. Elytral surface matte to somewhat shiny and iridescent	***traili* group**
–	Protibia with robust seta of dorsal margin approximately at half-length of outer margin, distance between robust seta and dorsoapical angle subequal to distance between robust seta and protibial insertion (Fig. 2d). Color variable, but elytra of mature specimens of most species with darker triangle medially at base (Fig. 1g, i). Most species with elytral surface very shiny, iridescent	***meizon* group (in part)**

### Description of species groups

### 1. N. josiahi

Taxon classificationAnimaliaColeopteraNoteridae

species group

708A15B4-9F34-570B-8828-57B076C4197C

#### Diagnosis.

**Figure 2.**
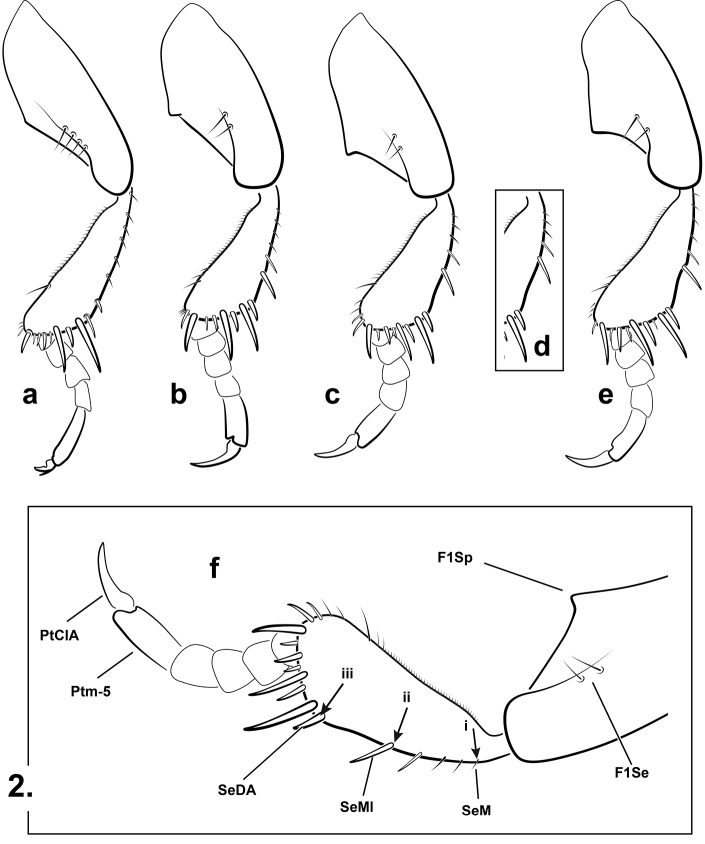
Representative prolegs of *Notomicrus* species groups (left proleg, anterior aspect) **a***josiahi* group (*N. josiahi*) **b***nanulus* group (*N. nanulus*) **c***meizon* group (*Notomicrus* sp.) **d***meizon* group, alternative setal spacing of dorsal (outer) protibial margin (*Notomicrus* sp.) **e***traili* group (N. cf. traili) **f** detail of structures of importance. F1Se = setae of anteroventral margin of profemur; F1Sp = protuberance of posteroventral margin of profemur; PtCA = anterior protarsal claw; Ptm-5 = protarsomere V; SeDA = first robust seta of dorsoapical angle; SeMl = robust seta at mid-length of anterodorsal margin of protibia; SeM = First seta of anterodorsal margin of protibia; arrows indicate points for relative lengths (see key): i = anteroapical angle, ii = robust seta at mid-length, iii = first seta of marginal row.

**Figure 3.**
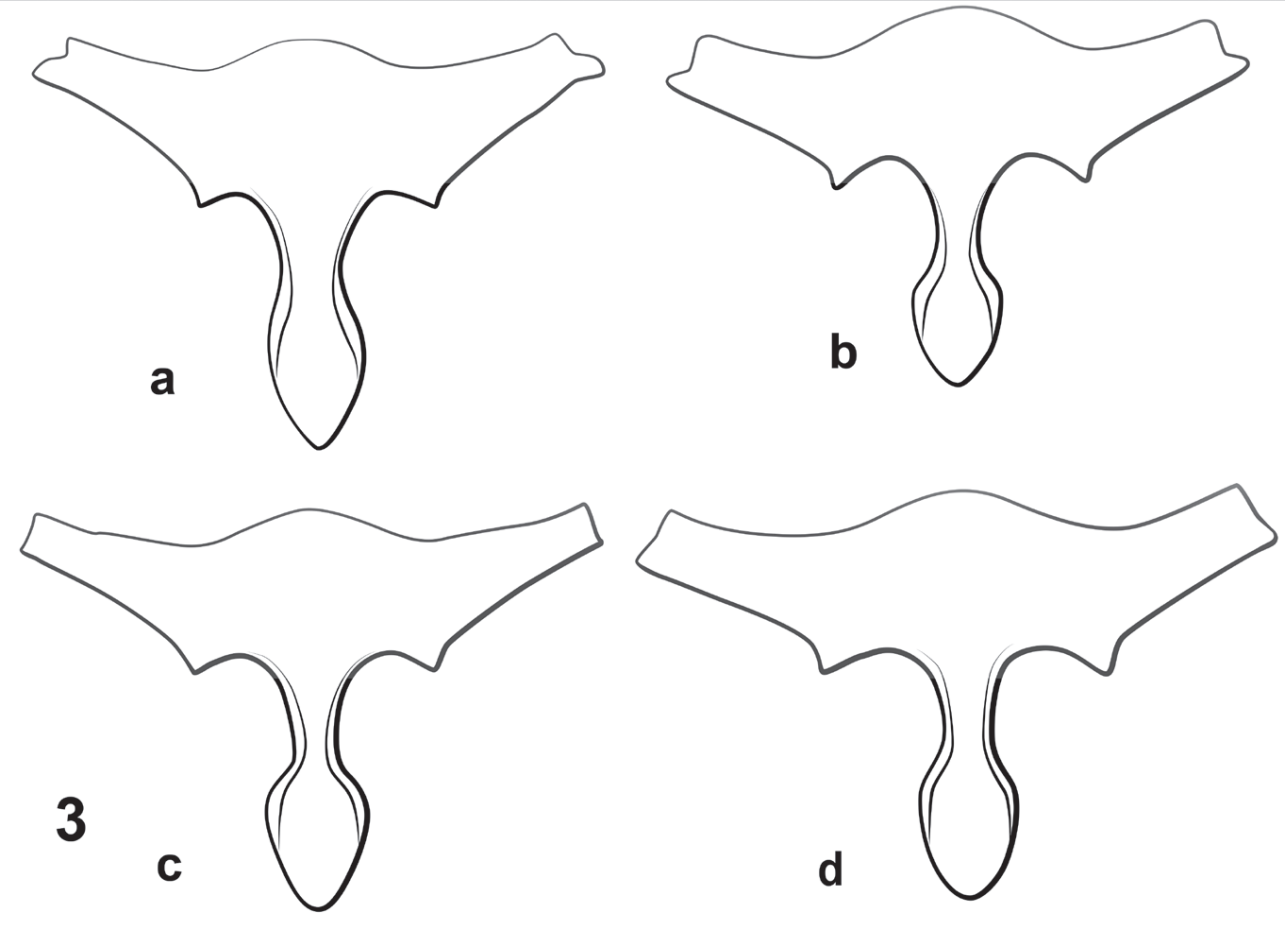
Representative prosterna of *Notomicrus* species groups **a***N. josiahi* group (*N. josiahi*) **b***N. nanulus* group (*N. nanulus*) **c***N. meizon* group (N. sp. nr. malkini) **d***N. traili* group (N. cf. traili).

The *josiahi* group is diagnosed by the following combination of characters. Dorsal (outer) margin of protibia without notable robust seta at or near mid-length (Fig. 2a). Body form elongate, strongly, but regularly attenuated posteriorly. Eyes large relative to head capsule. Elytra with notable contrasting dark and light colors (Figs 5, 6); shiny and iridescent; microsculpture very fine. Prosternal process narrow (Fig. 3a). Protibiae elongate, with penultimate dorsal seta only slightly longer than others on dorsal margin (Fig. 2a); males with profemur lacking notable protuberance on posteroventral margin (Fig. 2a), only weakly angulate at mid-length; protarsal claws very small, distinctly less than half the length of protarsomere V (Figs 2a, 8, 9), not distinctly larger than female claws, anterior protarsal claw bifurcate, with small dorsal spur (Figs 8, 9). The large eyes, elytral color pattern and coloration and characters of the protibiae, make this species group easily distinguishable from others. There are only two species known.

### Notomicrus josiahi

Taxon classificationAnimaliaColeopteraNoteridae

Miller, 2013

3E45D83F-C703-5A22-9286-FDDECF948067

**Figure 4.**
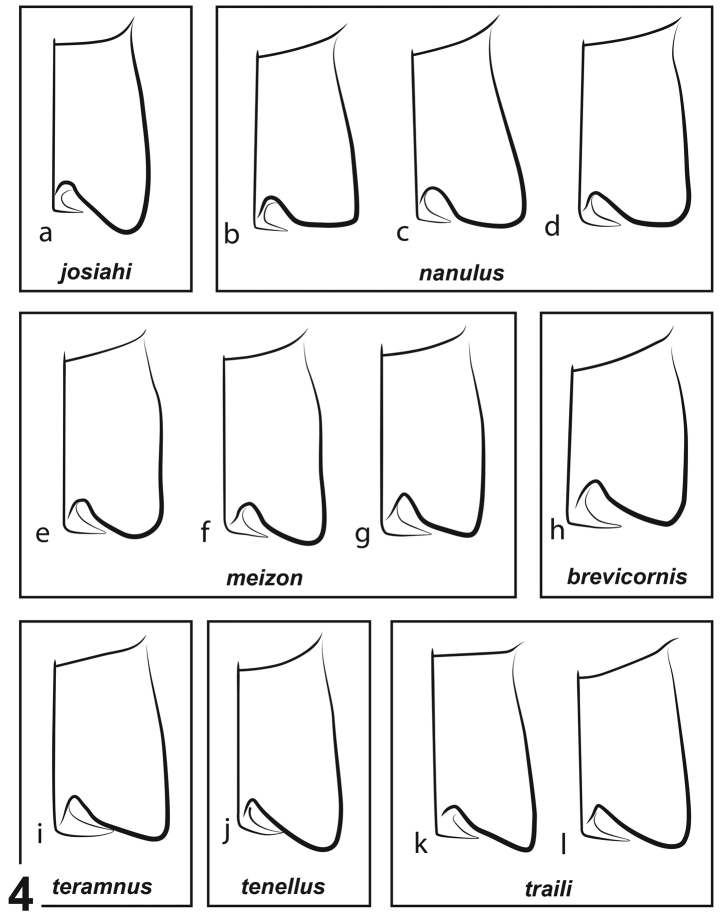
Representative noterid platforms of *Notomicrus* species groups (left side, ventral aspect). Names in boxes indicate species groups or species **a***N. josiahi***b***N. nanulus***c***N. sharpi***d***N.* sp. (nr. chailliei) **e**N. sp. nr. meizon**f***N. meizon* (paratype) **g**N. sp. nr. malkini**h***N. brevicornis* (female syntype) **i***N. teramnus* (female paratype) **j***N. tenellus* (Indonesia) **k***N. sabrouxi* (female paratype, sketched from Manuel (2015:518) **l***N. petrareptans*.

**Figure 5.**
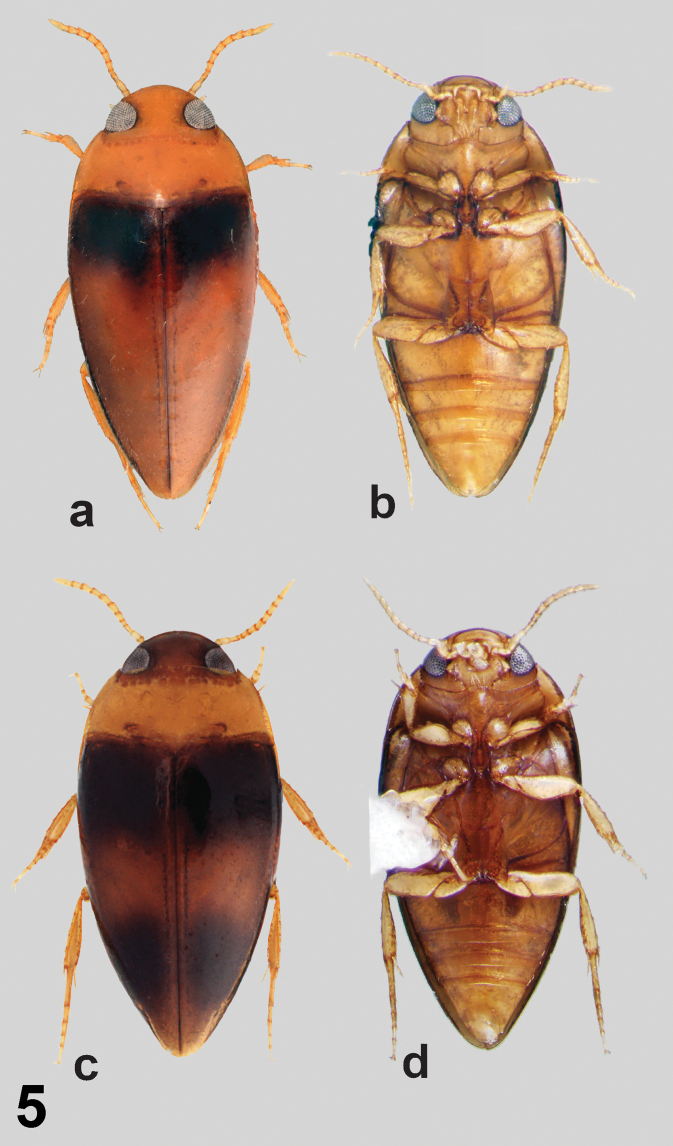
*Notomicrus josiahi* species group, dorsal and ventral habitus **a, b***Notomicrus josiahi* Miller, 2013 (paratype) **c, d***Notomicrus interstinctus* sp. nov. (paratype).

**Figures 6, 7.**
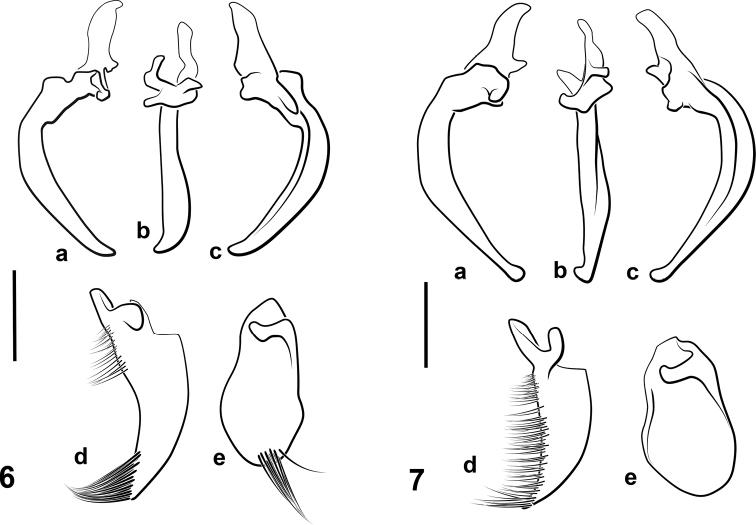
Aedeagi of *josiahi* species group **6***N. josiahi***7***Notomicrus interstinctus* sp. nov. **a** median lobe, left lateral aspect **b** median lobe, dorsal aspect **c** median lobe, right lateral aspect **d** left lateral lobe, medial surface/aspect **e** right lateral lobe, medial surface/aspect. Scale bars: 100 µm

**Figures 8, 9.**
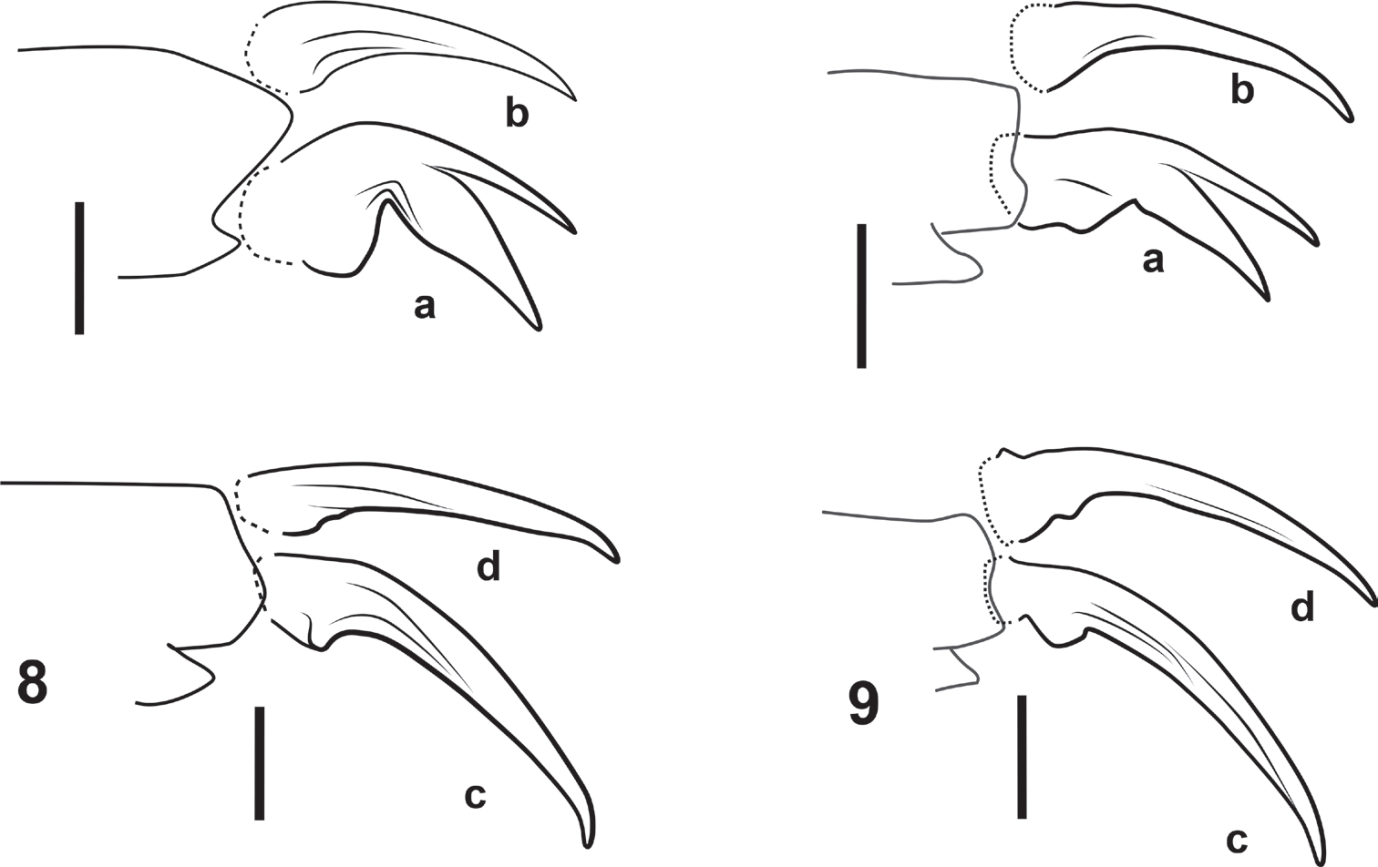
Pro- and mesotarsal claws of *josiahi* species group **8***N. josiahi***9***N. interstinctus* sp. nov. **a** anterior protarsal claw **b** posterior protarsal claw **c** anterior mesotarsal claw **d** posterior mesotarsal claw. All anterior aspect. Scale bars: 25 µm.

Figs 2a , 3a , 4a , 5a, b , 6 , 8

Notomicrus josiahi Miller, 2013: 244; Holotype: MIZA

#### Type locality.

Venezuela, Amazonas State, Communidad Caño Gato, Rio Sipapo, 4°58.838'N, 67°44.341'W.

#### Material examined.

***Paratypes***: “VENEZUELA: Amazonas State/ 4°58.845'N, 67°44.341'W, 100 m/ Communidad Caño Gato on Rio/ Sipapo; sandy stream; 7.i.2006; AS-06-016; leg. A.E.Z. Short” [White label, typed print] (1 female ex. SEMC); “VENEZUELA: Amazonas State/ 4°58.845'N, 67°44.341'W, 100 m/ Communidad Caño Gato on Rio/ Sipapo; 16.i.2009; leg. Short,/ Miller, Camacho, Joly, & Garcia/ VZ09-0116-01X; along stream” [White label, typed print] (1 male, 2 females ex. SEMC) All paratypes with white barcode label with the following numbers and “KUNHM-ENT”: “SM0843570” “SM0831496” “SM0842848” “SM0843672”; all paratypes with “PARATYPE/ *Notomicrus josiahi*/ Miller, 2013” [Blue label with black border, typed print].

#### Other material.

Venezuela: Amazonas State, 4°58.845'N, 67°44.341'W, 100 m, Communidad Caño Gato on Rio Sipapo; 16.i.2009; leg. Short, Miller, Camacho, Joly, & Garcia/ VZ09-0116-01X; along stream (64 males and females ex. SEMC).

#### Measurements.

TL = 1.46–1.69 mm (mean = 1.59 mm, SD. = 0.058, males = 1.46–1.69 mm, male mean = 1.58, SD. = 0.069, females = 1.55–1.68 mm, female mean = 1.62, SD. = 0.036); TLPn = 1.33–1.53 mm (mean = 1.44, SD. = 0.045, males = 1.33–1.49 mm, females = 1.43–1.53 mm); GW = 0.68–0.78 mm (mean = 0.74 mm, St. Dev. = 0.025, males = 0.68–0.78 mm, females = 0.72–0.78 mm); HW = 0.40–0.45 mm (mean = 0.42 mm, SD. = 0.014, males = 0.40–0.43 mm, females = 0.42–0.45 mm); EW = 0.16–0.19 mm (mean = 0.175 mm, SD. = 0.01, males = 0.16–0.17 mm, females = 0.17–0.19 mm); TL/GW = 1.99–2.31 (mean = 2.16; SD = 0.070; males = 1.99–2.31, females = 2.13–2.22); HW/EW = 2.21–2.53 (mean = 2.39, SD = 0.083, males = 2.41–2.53, females = 2.21–2.44).

#### Diagnosis.

*Notomicrus josiahi* can be diagnosed by the following combination of characters: (1) Size large TL = 1.46–1.69 mm; (2) elytron with strongly darkened region in anterior 1/3^rd^, contrasting against brownish-yellow of rest of elytron (Fig. 5a); (3) Eyes very large relative to head capsule (HW/EW = 2.21–2.53; males 2.41–2.53, females 2.21–2.37); (4) aedeagus as in Fig. 6, median lobe expanded on right side in dorsal or ventral aspect, weakly attenuated to apex from mid-length in lateral aspect, with apex curved dorsolaterally to the left, left lateral lobe with dense tuft of setae at apex, few setae along dorsal margin and sparse tuft near base; right lateral lobe with small tuft of setae at apex; (5) pro- and mesotarsal claws as in Fig. 8, anterior protarsal claw strongly bent, bifurcate, with slender spur originating on dorsal margin where curved (Fig. 8a), ventral margin strongly expanded ventrally near base.

#### Re-description.

**Males.** Body elongate-oval, attenuated posteriorly (Fig. 5a), TL/GW = 1.99–2.31 lateral outline of elytra and pronotum continuous in dorsal aspect; regularly curved to head, posteriorly evenly attenuated to elytral apex from point of greatest width; widest point just posterior to humeral angles of elytra, as in Fig. 5a.

***Color*.** Head, pronotum, venter and legs yellow; elytron dark brown to black in basal 1/3, darkened region extending posteriorly along elytral suture and contrasting against brownish-yellow color of posterior 2/3 of elytron (Fig. 5a); elytron with surface weakly iridescent. Venter and legs uniformly yellow (Fig. 5b).

***Structures*.** Eyes very large relative to head capsule (HW/EW = 2.35–2.53); antennae with length greater than greatest width of head. Prosternal process narrow, not strongly constricted between procoxae, with apex attenuated (Fig. 3a). Noterid platform with lateral margins subparallel (weakly convergent in posterior 2/3, convergent in anterior 1/3 (Fig. 4a); posterior lobes acute, angled, acutely rounded at apex. Profemur with loose comb of 3–5 stiff setae on anteroventral margin (Fig. 2a), posteroventral margin weakly angled at mid-length (Fig. 2a). Protibia elongate, dorsal and ventral margins weakly divergent distally in anterior aspect (Fig. 2a), anterodorsal margin with row of 6–7 stout setae, without distinctly larger seta near mid-length. Protarsi with adhesive discs on ventral surface of protarsomeres II and III, lacking disc on ventral surface of protarsomere I; protarsal claws as in Fig. 8a, b, subequal in length, small, length ca. 1/3 that of protarsomere V, anterior claw distinctly bifurcate in distal half, expanded basally, very sharply curved, posterior claw slender, weakly expanded basally, moderately curved. Mesotarsi with adhesive discs on ventral surface of protarsomere II only, lacking disc on ventral surface of protarsomere I; mesotarsal claws as in Fig. 8c, d, subequal in length, small, length slightly greater than that of protarsal claws, slender, weakly expanded at base and weakly curved.

***Sculpture*.** Dorsal surface of head with microsculpture very weakly impressed, microreticulation very fine, meshes mostly indistinct; micropunctation nearly indistinct. Pronotum with microsculpture similar to that of head, microreticulation fine; with scattered punctation near base and lateral margin, lateral punctures moderately dense, some with very fine setae. Elytron with microsculpture weakly impressed, microreticulation very fine, nearly indistinct; with punctation sparse in anterior half, with fine punctures along lateral margin and along discal row, with very few to no punctures between discal row and elytral suture, punctate in posterior half, punctures fine, many with very fine setae; discal row composed of fine and irregularly scattered punctures, denser posteriorly, lateral row similar to discal row but more sparse; micropunctation present, evenly scattered. Noterid platform and metaventrite surface with microsculpture weakly to moderately impressed, very fine, meshes of microreticulation nearly indistinct, cells transversely elongated.

***Aedeagus*.** Aedeagus as in Fig. 6. Median lobe in lateral aspect gradually curved from base to apex, dorsal and ventral margins subparallel, converging at apex; apex acute, sharply curved, in ventral aspect subapically expanded and curved to left (Fig. 4a–c). Left lateral lobe in lateral aspect elongate, curved dorsally, with dense tuft of setae at apex (Fig. 6d). Right lateral lobe in lateral aspect oval; apex rounded with small tuft of setae in apical cleft (Fig. 6e).

**Females.** As males, except eyes slightly smaller than in males (HW/EW females = 2.21–2.39); profemur with posteroventral margin smooth, lacking weak angle at mid-length; pro and mesotarsomeres unmodified, slender, lacking adhesive discs; pro and mesotarsal claws unmodified, claws of respective tarsi subequal in length, slender, weakly curved.

#### Variation.

As this species is known from only a single series, it is difficult to assess the degree of intraspecific variation. However, some variation was observed in the relative lightness or darkness in coloration of the individuals, with some brighter in color, more yellow, and others darker in color, more brownish yellow. The darkened region of the elytra also varied somewhat, occupying 1/4 to greater than 1/3 of the basal region of the elytron.

#### Differential diagnosis.

*Notomicrus josiahi* is among the most easily distinguished species of *Notomicrus* by the combination of the large eyes, color pattern, shape of male protarsal claws and of male aedeagus. Superficially, *N. josiahi* is similar to some species of the *N. meizon* group in color, wherein *N. meizon* Guimarães & Ferreira-Jr, 2019, *N. malkini* Young, 1978 and other undescribed species are also darkened at the base of the elytra. However, in *N. josiahi*, this darkened area is better defined with the posterior border less oblique, thus expanding more completely over the humeral angles of the elytron. More distinctly, *N. josiahi* differs from these and other species by the much larger eyes and bifurcate protarsal claws (in males), which to date, has only been observed in *N. interstinctus*, *N. brevicornis* and the *tenellus* group. Among all other species of *Notomicrus*, the aedeagus of *N. josiahi* is distinct, with the right lateral lobe rounded and bearing a small tuft of setae at apex, rather than without setae, as in all other neotropical species.

#### Distribution.

**Figure 10.**
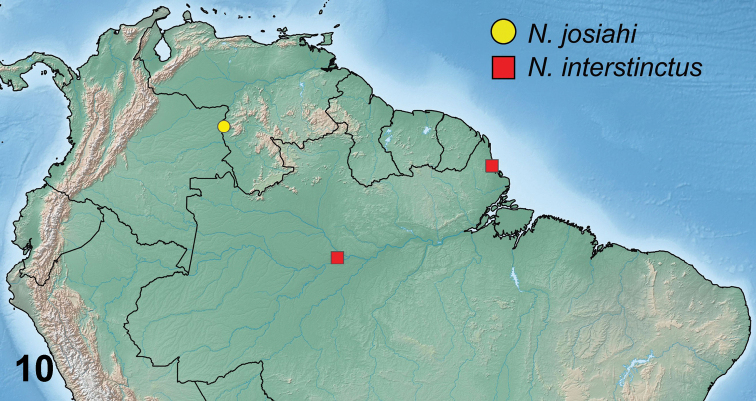
Distribution map of *josiahi* group species.

Known only from Venezuela (Fig. 10).

#### Ecology.

**Figure 11.**
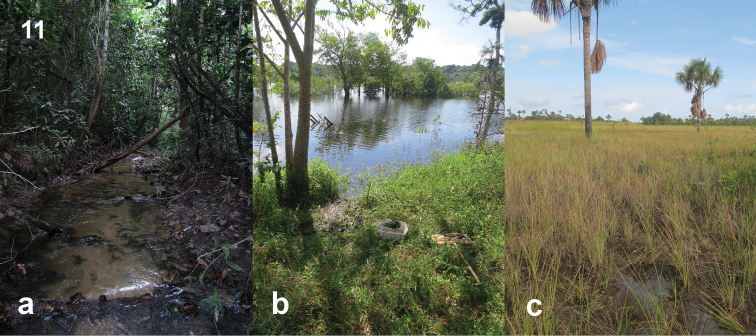
Habitats of *N. josiahi* group species **a** Type locality of *N. josiahi*, Venezuela: Amazonas (collection code VZ09-0116-01A); Localities of *N. interstinctus***b** Brazil, Amazonas (collection code BR17-0609-01A) **c** type locality, Brazil, Amapá (collection code BR18-0722-01A).

This species has been collected from only a single locality, from the margins of a small, sandy stream (Fig. 11a).

### Notomicrus interstinctus sp. nov.

Taxon classificationAnimaliaColeopteraNoteridae

28784C87-2C30-5AB7-8A87-716F9A280F82

http://zoobank.org/9098C43C-66D4-4245-8F3F-5A4F7FB62549

Figs 5c, d , 7 , 9

#### Type locality.

Brazil: Amapá, Calcoene, 2.50019, -50.97712.

#### Material examined.

***Holotype*, male**: “BRAZIL: Amapá: Calcoene/ 2.50019°, -50.97712°; 5 m/ Colcoene (1 km W) on BR-156/ 22.vii.2018; leg. Short; Marshy/ savannah; BR18-0722-01A” [White label, typed print] “HOLOTYPE/ *Notomicrus/ interstinctus*/ Baca & Short, 2020” [Red label, black border, typed print] (ex.INPA). ***Paratypes***: Same data as holotype, except with “PARATYPE/ *Notomicrus/ interstinctus*/ Baca & Short, 2021” [Blue label, black borders, typed print] (4 males, 5 females exs. SEMC, INPA); Brazil: Amazonas, Manacapuru Municipality, -3.23037, -60.64269, 35 m, 9.vi.2017, leg. Benetti, margin of large marsh/river, lots of vegetation; BR17-0609-01A; with “PARATYPE/ *Notomicrus/ interstinctus*/ Baca & Short, 2021” [Blue label, black borders, typed print] (3 males, 6 females exs. SEMC, INPA).

#### Measurements.

TL = 1.50–1.63 (Holotype = 1.50 mm, mean = 1.56 mm, SD. = 0.045, males 1.50–1.63 mm, females 1.50–1.63 mm); TLPn = 1.38–1.48 (Holotype = 1.40 mm, mean = 1.42 mm, SD = 0.039, males = 1.40–1.45 mm, females = 1.38–1.48 mm); GW = 0.72–0.80 mm (Holotype = 0.72 mm, mean = 0.75 mm, SD. = 0.027, males = 0.72 mm–0.76 mm, females = 0.73–0.80 mm; HW = 0.41–0.45 mm (Holotype = 0.41 mm, mean = 0.43 mm, SD. = 0.013, males = 0.41–0.42 mm, females = 0.42–0.45 mm); EW = 0.18–0.22 mm (Holotype = 0.18 mm, mean = 0.20 mm, SD. = 0.013, males = 0.18–0.19 mm, females = 0.19–0.22 mm), TL/GW = 1.99–2.26 (Holotype = 2.08, mean = 2.07, SD. = 0.074, males = 2.06–2.26, females = 1.98–2.11); HW/EW = 2.04–2.33 (Holotype = 2.28, mean = 2.19, SD. = 0.088, males = 2.16–2.33, females = 2.04–2.26)

#### Diagnosis.

*Notomicrus interstinctus* can be diagnosed by the following combination of characters: (1) Size large TL = 1.53–1.63 mm; (2) elytron dark with contrasting yellow band at mid-length, apices yellow (Fig. 5c, d); (3) eyes very large relative to head capsule (HW/EW = 2.04–2.33; Fig. 5c); (4) aedeagus as in Fig. 7; median lobe not broadly expanded on right side in dorsal aspect, attenuated to apex in lateral aspect, with small, round apical club oriented laterally to left; left lateral lobe with row of setae along entire dorsal margin, only somewhat denser at apex; right lateral lobe glabrous, without small tuft of setae at apex; (5) protarsal claws as in Fig. 9a, b; anterior protarsal claw strongly bent, bifurcate, branching at mid-length, ventral margin somewhat expanded ventrally near base.

#### Description.

***Holotype*.** As described for *N. josiahi*, except the following. Size large, TL = 1.53 mm. Body very broad, elongate-oval, strongly attenuated posteriorly, TL/GW = 2.08; lateral outline of elytron evenly and gradually curved to apex from point of greatest width, as in Fig. 5c, d.

***Color*.** Dorsal surface of head brown, lighter near clypeus; pronotum yellow; elytron dark, nearly black in anterior and posterior thirds, with lighter contrasting brownish-yellow transverse band near mid-length of elytron, elytral apex also lighter, brownish-yellow; elytron with surface moderately iridescent. Ventral surface of head and prosternum light brownish-yellow; rest of venter yellowish-brown; legs yellow.

***Structures*.** Eyes large relative to head capsule (HW/EW = 2.28). Posterior lobes of noterid platform with angles acute, apices rounded (as in Figs 3a, 5d). Pro- and mesotarsal claws as in Fig. 9.

***Sculpture*.** Elytron with punctation as described in *N. josiahi*, but denser overall and less restricted to posterior half, with punctures along lateral margin and puncture rows more widely distributed and denser.

***Aedeagus*.** Aedeagus as in Fig. 7. Median lobe in lateral aspect, strongly curved at base, distally weakly curved, nearly straight; dorsal and ventral margins subparallel to mid-length, then attenuated to apex; apex with small club, sharply bent dorsally and left; left lateral lobe in lateral aspect, elongate, dorsal margin curved with dense row of fine setae (Fig. 7d). Right lateral lobe in lateral aspect oblong, rounded distally.

**Females.** As males, except eyes slightly smaller (HW/EW = 2.04–2.16); profemur with posteroventral margin smooth, lacking weak angle at mid-length; pro- and mesotarsomeres unmodified, slender, lacking adhesive discs; pro- and mesotarsal claws unmodified, claws of respective tarsi subequal in length, slender, weakly curved.

#### Variation.

The most notable variation was in size and color, with some specimens darker overall than others, with the yellow bands sometimes smaller.

#### Differential diagnosis.

*Notomicrus interstinctus* is easily distinguished by the combination of large eyes and elytral color pattern, darkened in anterior and posterior thirds with a yellow transverse band. This color pattern is unique among known species of *Notomicrus*. This species is also unusual in that it is one of the few known species (along with *N. brevicornis*, *N. josiahi* and members of the *tenellus* group), with males that present bifurcated anterior protarsal claws. The aedeagus, color pattern and more subtly the denser punctation, easily differentiates this species from *N. josiahi*. The aedeagus of *N. interstinctus* is similar to that of the *N. traili* group with the median lobe attenuated and the apex enlarged and bent in a left-dorsal direction, but other external characters, such as the color pattern, tarsal claws and large eyes, readily distinguish this species from the *traili* group. The elytral punctuation is somewhat similar to that of some members of the *N. meizon* group, being somewhat densely punctate posteriorly, with punctures fine, but the aforementioned combination of characters will differentiate *N. interstinctus* from these species as well.

#### Etymology.

*Notomicrus interstinctus* sp. nov. derives its name from the Latin adjective *interstinctus*, meaning checkered or variegated. This refers to the color pattern of the elytra of this species. It is treated as an adjective in the nominative singular.

#### Distribution.

Known from northern Brazil, Amazonas and Amapá states (Fig. 10).

#### Ecology.

The species seems to be a generalist in terms of habitat, but seems to prefer vegetated environments. It was collected from a very shallow open marshy area (Fig. 11c) in the Brazilian state of Amapá and the vegetated margins of a river in Amazonas (Fig. 11b).

#### Taxonomic comments.

*Notomicrus interstinctus* appears very similar to specimens misidentified as *N. traili* Sharp, 1882 by Guimarães and Ferreira-Jr (2019). This was due to the similarities of the size, punctation and shape of the aedeagus. The records from this work would potentially add to the distribution above, as most appear to be from the same regions of the Amazon Basin as the Amazonas specimens. Verification will be needed to confirm these individual records and these are not formally attributed to *N. interstinctus* here. Observations of the lone female syntype of *N. traili* (NHM) indicate that the species is as described by Young (1978), with males attributable to *N. traili*, appearing as in Fig. 2, with the head and elytra brown, without a pattern.

### 2. Notomicrus nanulus

Taxon classificationAnimaliaColeopteraNoteridae

species group

6462B419-D197-5875-B530-E2A73C87E3E7

#### Diagnosis.

Members of this species group are most easily identified by their (1) monotone brown elytral color (Fig. 1a–c); (2) rounded, oval body shape; (3) rounded posterior lobes of the noterid platform (Fig. 4b–d); and (4) by their coarser microsculpture, consisting of isodiametric cells, appearing scale-like, rather than as a finer mesh of transversally elongated cells. This latter character is best viewed in light reflecting off the elytra. Even in species with finer variants of the cell-like microsculpture (e.g. *N. sharpi*), there is no iridescence. Punctation is largely indistinct, except for the discal series and sometimes sporadic punctures posteriorly.

Members of the *N. nanulus* group present a combination of characters that are variably shared with *N. brevicornis*, *N. teramnus* and the members *N. tenellus* group. This pattern, in tandem with our phylogenetic understanding, for example, *tenellus* group being sister to all other *Notomicrus* (Baca and Short 2020), suggest that *N. nanulus* species are united by a combination of characters that are plesiomorphic at some level within the genus. All share similar microsculpture consisting of isodiametric cells, often appearing scale-like to some degree. However, the *N. nanulus* group is distinguished from the *N. tenellus* group by the more rounded body outline (Fig. 1a–c, j) and from the *N. tenellus* group and *N brevicornis* by the shape of the noterid platform, with the *nanulus* group presenting posterior lobes that are rounded or squared (Fig. 4b–d). The rounded/squared lobes of the noterid platform character distinguish the *nanulus* group from *N. teramnus* (Fig. 4i) also, but more subtly. The *nanulus* group also typically presents a noterid platform with more longitudinally-elongated proportions than *N. brevicornis* (Fig. 4h). Males of the *N. nanulus* group present unbifurcated anterior protarsal claws, unlike *N. brevicornis* and the *N. tenellus* group. The aedeagi of known males of the *N. nanulus* group are easily distinguished; see Young (1978) and Manuel (2015).

#### Composition.

*N. chailliei* Manuel, 2015; *N. femineus* Manuel, 2015; *N. huttoni* Young, 1978; *N. nanulus* (LeConte, 1863); *N. sharpi* Balfour-Browne, 1939.

#### Identification resources.

Young (1978); Manuel (2015).

#### Remarks.

Future work on this group may prove difficult as many species are collected with high ratios of females to males. An example was *N. femineus* Manuel, 2015, in which extensive collecting yielded only females, raising the possibility of parthenogenetic reproduction. Personal observations indicate that multiple undescribed species are represented by females only. We note that females of this group can be especially difficult to distinguish and are often misidentified as *N. brevicornis* (see comments on *N. brevicornis*, below). *Notomicrus teramnus* is a potential member of this species group based on color, shape and microsculpture, but is treated separately in the key, pending further investigation (see *insertae sedis* species below).

### 3. Notomicrus meizon

Taxon classificationAnimaliaColeopteraNoteridae

species group

BADEB464-181C-59D2-ADC6-E2294A07D016

#### Diagnosis.

Non-teneral specimens of this group tend to have the following combination of characters: (1) triangular pigmented area medially on the base of the elytra (Fig. 1g–i), similar to *N. josiahi*, but not as prominent and not always discernible in some populations or in teneral specimens; (2) dense, fine punctures bearing short setae on the posterior half of the elytra and sometimes extending far anteriorly (not as coarse as in members of the *traili* group); (3) microreticulation variably impressed, consisting of fine mesh-like reticulation; often iridescent; (4) posterior lobes of noterid platform with squared or rounded angles (Fig. 4e); if posterior angles of noterid platform more acute (Fig. 4f, g), protibia presents robust seta of outer margin approximately at half-length of outer margin, distance between robust seta and dorsoapical angle subequal to distance between robust seta and first seta from protibial insertion (Fig. 2d).

#### Composition.

*N. malkini* Young, 1978; *N. meizon* Guimarães & Ferreira-Jr, 2019.

#### Identification resources.

Young (1978); Guimarães and Ferreira-Jr (2019).

#### Remarks.

The *meizon* group is sometimes difficult to discern from the *traili* group, as the differences amongst diagnostic characters can be subtle. The darkened basal area of the elytra in the *meizon* group is helpful, but investigators may find great difficulty in diagnosing teneral members of this group, which often lack the pigmented triangular area on the elytra. It is important to note that this darkened area is truly pigmented, not just darker in appearance due to the folding of the wings under the elytra as often happens in lighter colored species (as in Fig. 1f, j, k). Fortunately, males of most individual species of the *meizon* group are easy to identify by their aedeagi in combination with other characters, such as tarsal claws. The male median lobes of the *meizon* group species are usually (but not always) very irregularly shaped (for example, see Young 1978 and Guimarães and Ferreira-Jr 2019). The aedeagus of most species of the *traili* group appear similar to Fig. 8, with a small club at apex, often hooked to the left (see Young 1978; Manuel 2015; Baca and Short 2018). Additionally, males of *meizon* species often present notable unequal lengths between the anterior and posterior protarsal claws. These are usually subequal in length in the *traili* group.

### 4. Notomicrus traili

Taxon classificationAnimaliaColeopteraNoteridae

species group

217EC1DA-AD8C-5E33-A05E-4C69B09D2C48

#### Diagnosis.

Non-teneral specimens of this group tend to have the following combination of characters: (1) lacking triangular pigmented area on the medial base of the elytra (Fig. 1d–f), lacking maculae; elytra with uniform shades of tan or brown; (2) irregular setose punctures in posterior half of elytral surface with density variable, increasingly coarse if extending on to anterior half of elytron; (3) microreticulation variably impressed, consisting of fine mesh-like reticulation; matte to shiny; elytral surface sometimes somewhat iridescent; (4) posterior lobes of noterid platform with angles acute (Fig. 4k, l); (5) protibia as in Fig. 2e, with robust seta of outer margin at ca. 2/3 length of margin, distance between robust seta and dorsoapical angle distinctly less than distance between robust seta and first seta from protibial insertion.

#### Composition.

*N. gracilipes* Sharp, 1882; *N. petrareptans* Baca & Short, 2018; *N. reticulatus* Sharp, 1882; *N. sabrouxi* Manuel, 2015; *N. traili* Sharp, 1882.

#### Identification resources.

Young (1978); Manuel (2015); Baca and Short (2018).

#### Remarks.

Species of the *traili* group are difficult to discern and constitute a widespread species complex (see Baca and Short 2020). Personal observations coupled with the phylogenetic reconstructions of Baca and Short (2020) show that the diagnostic power of the dorsal punctation (see Young 1978) is unreliable, with multiple clades within the complex sharing similar patterns of punctation; for example, the pattern of punctation attributed to *N. gracilipes* by Young (1978) arises in multiple places within the complex. The group will require careful taxonomic investigation. The members of the *traili* group can be difficult to distinguish from those of the *meizon* group, but mature members lack a pigmented triangular area at the base of the elytra and most males of the *traili* group have similarly-shaped median lobes of the aedeagus, distinct from the *meizon* group. See notes in remarks of *meizon* group above.

##### 5. *Insertae sedis* species

These species present characters combinations not found in other species groups. Both by presented character combination and even general *gestalt*, these are difficult to place with certainty. Molecular sequence data were unavailable for these species in the phylogenetic reconstruction of Baca and Short (2020). In particular, the species listed here both exhibit body shape, color, microsculpture and sparse punctation that would place them in the *N. nanulus* species group. However, in comparison with the *N. tenellus* species group, the sister to all New World taxa, several of these characters appear plesiomorphic in *Notomicrus*, making it difficult to discern their likely relatives from morphology alone.

### N. brevicornis

Taxon classificationAnimaliaColeopteraNoteridae

Sharp, 1882

5BDD9BCE-E76C-5083-8A53-01EFA158544B

Figs 1k , 12

#### Material examined.

***Syntypes***: Male specimen on small rectangular card, “♂” is drawn around genitalia and other parts, prosternal process flanks the specimen. “Boa Sorta Nov./ Sahlberg 1850” [small rectangular label, handwritten], “Sharp Coll/ 1905-313” [small rectangular label, typed], “Notomicrus/ brevicornis Ind. typ./ D.S.” [small rectangular label, handwritten] “SYN/ TYPE” [small circular label with blue border, printed] (ex. NHM); female specimen on rectangular card, “S. America/ Brazil.” [small rectangular label with blue line across, typed], “Sharp Coll/ 1905-313.” Small rectangular label, typed], “Boa Sorta Nov./ Sahlberg 1850” [small rectangular label, handwritten], “Type 470/ Notomicrus/ brevicornis/ Boa Sorta” [rectangular label, handwritten], “SYN/ TYPE” [small circular label with blue border, printed], “TYPE” [small circular label with red border, printed], (ex. NHM); female specimen disarticulated on large card, “S. America/ Brazil.” [small rectangular label with blue line across, typed], “Boa Sorta Nov./ Sahlberg 1850” [small rectangular label, handwritten], “Notomicrus/ brevicornis, Sharp./ Co-type.” [rectangular label, handwritten], “SYN/ TYPE” [small circular label with blue border, printed], (ex. NHM); female specimen on small rectangular card, “Co-/ type” [small circular label with yellow border, printed], “S. America/ Brazil.” [small rectangular label with blue line across, typed], “Sharp Coll/ 1905-313.” Small rectangular label, typed], “Notomicrus/ brevicornis, Sharp./ Co-type.” [rectangular label, handwritten] “SYN/ TYPE” [small circular label with blue border, printed] (ex. NHM). Note: this latter specimen also with small label “Not brevicornis/ maybe gracilipes?/ Manuel det. 2016”. See notes below.

#### Remarks.

*Notomicrus brevicornis* would otherwise appear to be a member of the *nanulus* group by the aforementioned characters. However, it differs by the more acute posterior angles of the noterid platform, a character shared with members of the *tenellus*, *josiahi* and *traili* groups. The male syntype presents a bifurcate anterior protarsal claw (as in fig. 8A), a character shared by the *josiahi* and *tenellus* species groups. With the Old World and New World taxa being reciprocally monophyletic (Baca and Short 2020) and the plesiomorphic appearance of these characters, we would speculate that this species is likely to be sister to one of the New World species groups.

**Figure 12.**
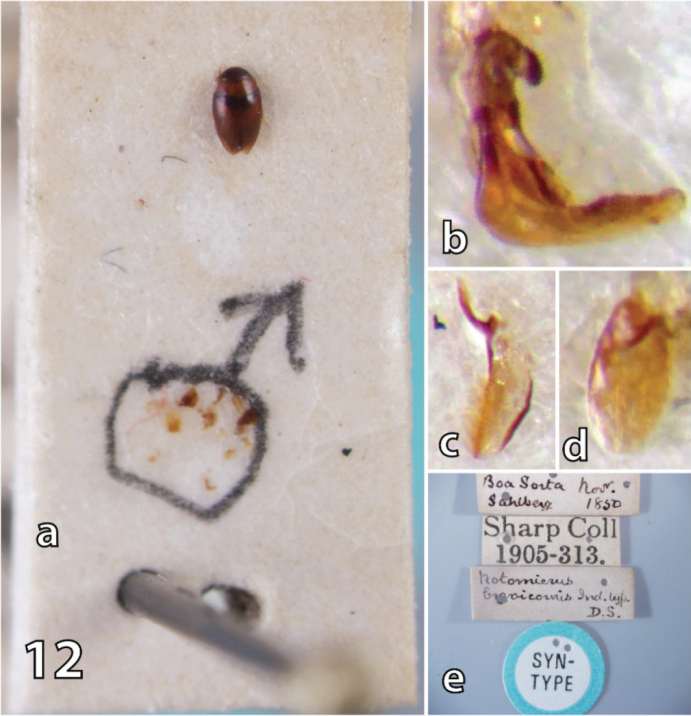
Card mount, aedeagus and labels of male syntype of *N. brevicornis***a***Notomicrus brevicornis* card mount, dorsal **b** median lobe lateral aspect **c** left lateral lobe, medial aspect **d** right lateral lobe **e** syntype labels.

Based on observation of the single male of the syntype series, it is suspected that Young (1978) based his description, key and illustration of the aedeagus of *N. brevicornis* on the male of a different species. First, the illustration in Young (1978) of the aedeagus of *N. brevicornis* does not match that observed in the syntype. Second, Young (1978: 288–289) describes *N. brevicornis* as being sexually dimorphic in elytral punctation, with males being more punctate than females. However, as noted by Sharp (1882: 261), there is very little dimorphism observed between males and females of the syntype series beyond characters of the tarsi. The punctation and sculpture are very weakly dimorphic, both sexes being almost entirely impunctate, except for the weak discal rows and a few scattered punctures near the elytral apex. The punctation is slightly less impressed in females, with discal rows slightly less prominent. The relative difference of punctation between the male and female syntypes of this species is so slight that splitting them up in the key as did Young (1978: 288, couplet 7) seems largely unnecessary, wherein the couplet describing females of *N. brevicornis* also closely describes the male syntype (Young 1978: 288). The specimens of the UMMZ, observed by Young, were not observed for this study, but the stated differences by Young (1978) and the grouping of males of *N. brevicornis* with *N. malkini* in Young’s (1978: 288) key call the identity of the depicted male in Young (1978) into question. Further adding to this suspicion is the fact that some male specimens attributable to *N. malkini* or other undescribed species of the *meizon* group in the FSCA were identified as *N. brevicornis* by Young (date of determination not recorded). For aiding in identification, we have included images of the male syntype, labels and aedeagus (Figs 1k, 12). One specimen of the syntype series appears to be of a different species than the others; likely it is a member of the *traili* species group. See last listed specimen and note in the examined syntype material above.

Personal observations show that many members of the *N. nanulus* group are misidentified as *N. brevicornis* in collections. This is no doubt due to the superficial similarities of *N. brevicornis* to members of the *nanulus* group and scarcity of males in the *nanulus* group. With that, there are likely inaccuracies in literature with respect to records and distributions.

### N. teramnus

Taxon classificationAnimaliaColeopteraNoteridae

Guimarães & Ferreira-Jr, 2019

2A7ADB32-3952-55F9-939B-63DAE62EBBA5

#### Remarks.

*Notomicrus teramnus* would also appear a member of the *nanulus* group, given the above-mentioned characters. An argument could be made that this is the case as it only appears to differ in the shape of the posterior lobes of the noterid platform being more angular than most species in the *nanulus* group. This species otherwise appears to lack characters that would unite it with other species groups, though this will require examination and/or phylogenetic investigation. We abstain from placing it as member of the *nanulus* group as *N. teramnus* is known only from a high elevation hygropetric habitat, which may present confounding morphological specialization. Aedeagal morphology is not here considered to be indicative of a particular placement, but the very unusual morphology of the aedeagus of *N. teramnus* (see Guimarães and Ferreira-Jr 2019) further raises questions of placement.

## Supplementary Material

XML Treatment for Notomicrus

XML Treatment for N. josiahi

XML Treatment for Notomicrus josiahi

XML Treatment for Notomicrus interstinctus

XML Treatment for Notomicrus nanulus

XML Treatment for Notomicrus meizon

XML Treatment for Notomicrus traili

XML Treatment for N. brevicornis

XML Treatment for N. teramnus
